# Egg and meat quality attributes of Tukong, a rumpled chicken breed in Indonesia

**DOI:** 10.1016/j.psj.2026.106730

**Published:** 2026-03-02

**Authors:** Aprilianna Putri Zahara Nafsina Luvita Sari, Yuli Arif Tribudi, Yasin Pradana Maulana, Maria Ulfah, Danung Nur Adli

**Affiliations:** a Department of Animal Production, Faculty of Animal Husbandry, Universitas Padjadjaran, Sumedang, Indonesia; b Departement of Animal Science, Faculty of Agriculture, Universitas Tanjungpura, Pontianak, 78124, West Kalimantan, Indonesia; c Department of Animal Product Technology, Faculty of Animal Husbandry, Universitas Padjadjaran, Sumedang, Indonesia; d Division of Animal Biosystematics and Ecology, Department of Biology, Faculty of Mathematics and Natural Sciences, IPB University, Bogor, 16680, West Java, Indonesia; e Smart Livestock Industry Study Programme, Department of Feed and Animal Nutrition, Faculty of Animal Science and Technology, Universitas Brawijaya, Malang, 65145, Indonesia

**Keywords:** Carcass quality, Egg quality, Fatty acids, Indigenous chicken, Rumpless phenotype

## Abstract

The Tukong chicken is a distinctive Indonesian local breed characterized by its rumpless phenotype; however, scientific information regarding its productive and nutritional attributes remains limited. This study evaluated the physical quality of Tukong chicken eggs, carcass characteristics, nutrient and fatty acid compositions of both eggs and meat under tropical environmental conditions. A total of 40 eight-week-old Tukong chickens were used in this research. Egg external and internal quality traits were measured individually, while proximate and fatty acid analyses were conducted via pooled samples and liquid chromatography-mass spectrometry (LC–MS).

During the 14-day study, ambient temperatures ranged from 26 to 34°C, and afternoon temperature-humidity index (THI) values frequently exceeded the severe heat stress threshold (>82). The tukong eggs were round (egg index 79.32%) and had good internal quality, with a Haugh unit of 71.73 (Grade A). An average yolk colour score is 10.43. Egg composition consisted of 11.23% protein, 13.98% fat, and 72.91% moisture, while the meat had 19.74% protein and 18.15% fat. Carcass yield reached 78.9% potentially associated with the rumpless phenotype. In summary, Tukong chickens maintain favourable egg quality nutritional profiles, despite exposure to moderate to severe heat stress, highlighting their potential as resilient local genetic resources.

## Introduction

The availability of local chicken breeds plays a crucial role in supporting food security and providing affordable animal protein, particularly in rural and low-income communities across developing countries. Compared with commercial broiler chickens or laying hens, indigenous chickens are more adaptable to harsh environments, demonstrate disease resistance, and require lower management inputs (Bettridge et al., 2018; Guarino Amato et al., 2022). These adaptive traits make local breeds valuable components of sustainable and low-input production systems. In addition to their resilience, their eggs and meat are often perceived by consumers to possess superior flavour and nutritional quality, resulting in higher cultural and market value. Consequently, increasing global attention has been directed towards the conservation and genetic improvement of indigenous poultry resources (Desta, 2021). A comprehensive understanding of the productive and nutritional characteristics of local breeds is essential for designing effective breeding strategies and maximising their contribution to community-based poultry systems. Characterizing egg and meat quality represents a fundamental step in assessing their commercial viability and long-term sustainability.

Among Indonesia’s native genetic resources, the Tukong chicken represents a distinctive local breed characterised by a rumpless phenotype, marked by the absence of tail feathers and the pygostyle (Tribudi et al., 2023). This morphological trait, which has been documented in only a limited number of breeds worldwide, is believed to result from specific genetic mutations affecting feather and skeletal development. Despite its uniqueness and regional cultural significance, scientific data describing the productive performance and nutritional composition of the Tukong chicken remains extremely limited. In particular, information regarding egg quality traits, carcass characteristics, nutrient composition, and fatty acid profiles is largely unavailable.

Egg quality assessment is a key approach for characterising poultry genotypes. External and internal parameters, including egg dimension (length and width), shape index, shell thickness, the Haugh unit, and yolk pigmentation, serve as indicators of structural integrity, freshness, and consumer preferences (Da Silva Neto et al., 2024). Furthermore, the proximate and fatty acid compositions of eggs provided important information regarding their nutritional value and potential functional benefits (Usturoi et al., 2025).

Similarly, evaluating carcass yield and meat composition is essential for assessing the commercial potential of indigenous breeds. Protein and lipid contents, moisture levels, and fatty acid profiles influence both sensory properties and health implications, particularly in relation to cardiovascular risk (Gomez et al., 2020). Notably, the potential association between the rumpless phenotype and carcass distribution, fat deposition, or metabolic characteristics has not yet been investigated. This represents a significant gap in current knowledge and limits the development of evidence-based conservation and utilisation strategies for this breed.

Therefore, the present study was conducted to systematically evaluate the physical quality of Tukong chicken eggs, characterize carcass attributes, and determine the nutrient and fatty acid compositions of both eggs and meat. We hypothesised that, despite its unique rumpless morphology, the Tukong chicken would exhibit favourable egg and meat quality traits comparable to other indigenous breeds, supporting its potential as a resilient and nutritionally valuable local genetic source.

## Materials and methods

### Ethical approval

All experimental procedures involving birds were conducted in accordance with institutional and national guidelines for animal care and use. The study protocol was reviewed and approved by the Research Ethics Committee of Universitas Brawijaya (Approval No. 155-KEP-UB-2022).

### Birds management

Tukong chickens were sourced from smallholder family farms distributed across ten locations. Only breed identity and age classification were used as selection criteria. After procurement, all birds were maintained under standardized management conditions throughout the experimental period. Three housing systems were applied according to the physiological stage. Chickens aged one month until sexual maturity were reared in colony cages (5 m²). For breeding purposes, mating cages were arranged at a male-to-female ratio of 1:2. Additionally, individual cages (40 cm × 50 cm × 50 cm) were used to house males and females separately when necessary to prevent aggressive interactions. All cages were semi-closed house systems equipped with rice husk bedding, which was replaced regularly when wet or contaminated. Birds were fed 110 g per head per day, divided into two feeding times. The diet consisted of a 60:40 mixture of commercial broiler feed and rice bran. The formulated ration contained 88.03% dry matter, 15.30% crude protein, 5.78% crude fibre, 0.50% calcium, 0.92% phosphorus, and 2,227 kcal/kg metabolizable energy, with a gross energy content of 4,012 kcal/kg. Drinking water was provided ad libitum.

**Fig. 1:**
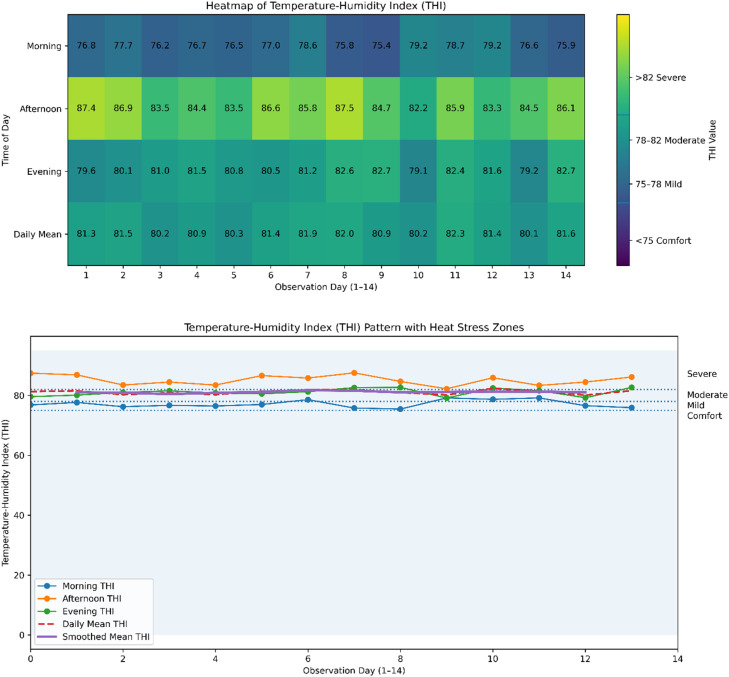
Heatmap and graph of temperature-humidity index during experiment.

The relative humidity in the morning was around 80-88%, while the temperature was mostly between 26 and 29°C. However, the afternoon temperature remained relatively constant at approximately 31-34°C, with relative humidity dropping to about 65–75%. In the evening, temperatures dropped to around 28–31°C with relative humidity hovering around 75–85%. The afternoon period was characterized by high ambient temperatures and relative humidity, resulting in the highest THI values. Conditions were milder in the morning but moderated by evening. Moderate to severe heat stress was caused by the diurnal shift, especially in the afternoon, for 14 consecutive days. Standardised colour values range from 70 to 90, with horizontal bands indicating the classification thresholds for heat stress (75 comfort; 75 to 78 mild; 82 to moderate; >82 severe). The severe stress threshold was frequently surpassed by the afternoon THI, as shown in Fig. 1.

### Egg sampling and handling

A total of 100 Tukong chickens were reared under uniform management conditions within a single conservation flock. Tukong chickens are considered a rare indigenous genetic resource with a limited breeding population. Therefore, a census-based sampling approach was adopted, in which all available Tukong chickens that laid eggs were included in the study rather than selecting a subset sample size.

Eggs were collected on the day of laying and stored at room temperature for 8 days to standardize egg age prior to evaluation. All physical egg quality traits were assessed individually for each egg (*n* = 100). For chemical analyses (including proximate composition and fatty acid profile), representative composite samples were prepared due to analytical requirements. Except for protein and fatty acid analysis, all evaluations were conducted on eggs stored for eight days.

### Chicken sampling and slaughter procedures

The Tukong chicken is a rare indigenous genetic resource with a limited breeding population. The sample size (*n* = 40; 20 males and 20 females) reflected all available birds within the defined age range

(6–8 months) that were accessible during the study period. Therefore, a census-based approach was applied rather than determining sample size through statistical power calculations. Because of the limited population size and conservation status of this breed, including all eligible individuals allowed us to maximise representativeness while minimising sampling bias. The study, therefore, can be interpreted as a baseline characterisation of this defined population rather than a population-wide inferential survey. The birds were slaughtered according to standard halal and welfare-compliant procedures. After exsanguination, feathers, head, neck, feet, and internal organs were removed, and carcasses were weighed. Meat samples were collected for proximate and fatty acid analysis.

### Egg quality, nutritional composition of eggs, and meat measurements

A total of 100 eggs were used for physical quality evaluation (*n* = 100). Eggs were collected once daily in the morning to ensure consistency and to minimize variation associated with the duration of laying. Second, after the eggs were cleaned and allowed to reach room temperature, egg weight (g) was determined via a calibrated digital scale with a precision of 0.001 g. Egg length and width (mm) were then measured via a digital calliper (China) to obtain accurate shell dimensions. For yolk weight (g), the yolk was carefully separated manually from the albumen and weighed individually via the same precision scale. Additionally, albumen weight (g) was calculated by subtracting the measured egg yolk and shell weights from the total egg weight. This indirect method is widely used because it provides a reliable estimate of albumen mass without disrupting its structure. The shell weight (g) was recorded after carefully removing and drying the shell membranes to ensure accurate measurement of the mineral shell alone. Shell membrane thickness (mm) was measured after manually separating the membranes from the calcified shell layer. Measurements were taken using a digital caliper with 0.001 mm precision, and the mean value was used for analysis. Moreover, yolk color was evaluated via a Roche Yolk Color fan (scale 1–15) (DSM-firmenich YolkFan™, Germany). Each yolk was compared against the standard color strips under natural light conditions, and the corresponding numerical score was recorded to reflect pigmentation intensity.

$$\mathrm{Yolk}\;\mathrm{Percentage}\;\left(\%\right)=\frac{\mathrm{Yolk}\;\mathrm{weight}}{\mathrm{egg}\;\mathrm{weight}}x100$$

$$\mathrm{Albumen}\;\mathrm{Percentage}\;\left(\%\right)=\frac{\mathrm{Albumen}\;\mathrm{weight}}{\mathrm{egg}\;\mathrm{weight}}x100$$

$$\mathrm{Egg}\;\mathrm{Shape}\;\mathrm{Index}\;=\frac{\mathrm{Egg}\;\mathrm{width}}{\mathrm{egg}\;\mathrm{length}}x100$$

$$\mathrm{Haugh}\;\mathrm{Unit}\;=100\log\left(H+7.57-1.7\;W^{0.37}\right)$$

Where *H* = albumen height (mm) and *W* = egg weight (g)

### Carcass trait measurements

The carcass yield was calculated as the ratio of the carcass weight to the pre-slaughter body weight. Live weight was recorded immediately before slaughter, whereas carcass weight was obtained after evisceration. The dressing percentage (%) was computed as:

$$\mathrm{Carcass}\;\mathrm{yield}=\frac{\mathrm{Carcass}\;\mathrm{weight}}{\mathrm{pre}-\mathrm{slaughter}}x100$$

### Visualisation and coding

Data processing and graphical visualisation were carried out using Python (version 3.10). NumPy was used for numerical computations, such as calculating the Temperature-Humidity Index (THI) using an established formula, while the pandas library was used for data handling and preprocessing. Matplotlib, a package, was used to visualise the data by adding shaded heat stress zones and threshold demarcations for easier interpretation.

## Results

### Egg quality

**Fig. 2:**
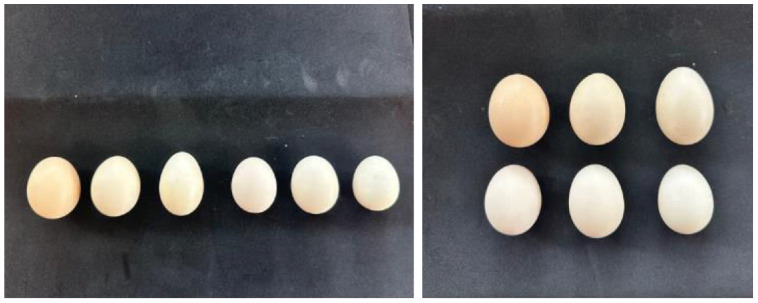
The appearance of Tukong chicken eggs.

The internal and physical characteristics of Tukong chicken eggs are presented in Table 1. Eggs exhibited an average length of 49.06 ± 2.39 mm and a width of 38.84 ± 0.94 mm, resulting in an egg index of 79.32 ± 3.85 (Table 1; Fig. 2), indicating a predominantly oval shape. Shell weight averaged 5.15 ± 0.74 g, with a relatively uniform thickness of 0.12 ± 0.001 mm. Yolk weight was 15.70 ± 1.40 g (Table 1), corresponding to 38.28 ± 2.26% of total egg weight, whereas albumen weight averaged 19.24 ± 1.35 g, accounting for 46.69 ± 1.46%. The mean Haugh Unit (HU) was 71.73 ± 4.66, indicating acceptable internal quality and albumen integrity. Yolk colour scored 10.43 ± 0.79 on the Roche scale, suggesting moderate pigmentation. Overall, Tukong chicken eggs demonstrated consistent structural uniformity and satisfactory internal quality traits.

**Table 1:** Quality of Tukong chicken eggs.

Parameter	Value
Egg length (mm)	49.06±2.39
Egg width (mm)	38.84±0.94
Egg index	79.32±3.85
Shell weight (g)	5.15±0.74
Shell membranes thickness (mm)	0.12±0.001
Egg yolk weight (g)	15.70±1.40
Egg yolk percentage (%)	38.28±2.26
Albumen weight (g)	19.24±1.35
Albumen percentage (%)	46.69±1.46
*Haugh Unit* (HU)	71.73±4.66
Egg yolk color	10.43±0.79

g – gram; mm – millimetre; % - percentage.

### Nutritional value of eggs and meat

Table 2 provides a summary of the mineral and proximate composition of Tukong chicken eggs and meat. Matrix compositions were distinct and pronounced. Meat had higher protein levels (19.74%) compared to eggs (11.23%). In the same way, meat had a higher fat content of 18.15% while eggs had an equivalent amount of 13.98%. Compared to meat, which had a lower moisture content of 56.40%, eggs were 7.9% more humid (72.91%). The ash content in meat was higher than that of eggs, with meat having a higher ash content (1.60%). The carbohydrate content of meat (4.12%) was approximately 4.2 times higher than that of eggs (0.98%). Mineral analysis revealed marked differences. The amount of calcium in eggs (74.67 g/100 g) was almost 4.8 times greater than that in meat (15.45 g/100 g), and the amount of phosphorus in eggs (273.72 g/100 g) exceeded that of meat (209.75 g/100 g) by approximately 30.5%. These observations highlight the distinct nutrient partitioning between eggs and muscle tissue in Tukong chickens.

**Table 2:** Nutritional quality of eggs and meat from Tukong chickens.

Parameters	Egg	Meat
Protein (%)	11.23±0.14	19.74±0.14
Fat (%)	13.98±0.06	18.15±0.02
Moisture content (%)	72.91±0.03	56.40±0.17
Ash (%)	0.90±0.01	1.60±0.07
Carbohydrates (%)	0.98±0.18	4.12±0.08
Calcium (mg/100 g)	74.67±0.88	15.45±0.19
Phosphorus (g/100 g)	273.72±0.55	209.75±0.83

g – gram, % - percentage.

### Fatty acid profile of Tukong chicken eggs

The fatty acid composition of Tukong chicken eggs is presented in Table 3. The lipid fraction was dominated by monounsaturated and saturated fatty acids. Oleic acid (C18:1 n-9) was the most abundant fatty acid (60,263.38 µg/g), followed by palmitic acid (C16:0; 37,915.03 µg/g) and linoleic acid (C18:2 n-6; 15,513.28 µg/g). Stearic acid (C18:0; 9,089.34 µg/g) and palmitoleic acid (C16:1 n-7; 7,652.90 µg/g) were also present in notable concentrations. Long-chain polyunsaturated fatty acids (PUFAs), including arachidonic acid (C20:4 n-6; 971.30 µg/g), eicosapentaenoic acid (EPA; 967.42 µg/g), and docosahexaenoic acid (DHA; 5,255.59 µg/g), were detected, indicating the presence of biologically relevant n-3 and n-6 fractions. Minor quantities of medium-chain fatty acids were observed. Overall, the fatty acid profile of Tukong chicken eggs was characterised by a predominance of oleic and palmitic acids, accompanied by measurable levels of essential polyunsaturated fatty acids.

**Table 3:** Fatty acid content of Tukong chicken eggs.

No	Code	Fatty Acid	Concentration (µg/g)
1	C_14:0_	*Myristic acid*	664.56
2	C_14:1 n-5_	*Myristoleate acid*	390.97
3	C_15:0_	*Pentadecanoic acid*	143.09
4	C_16:0_	*Palmitic acid*	37,915.03
5	C_16:1 n-7_	*Palmitoleic acid*	7,652.90
6	C_17:0_	*Margaric acid (Heptadecanoic acid)*	209.75
7	C_18:0_	*Stearic acid*	9,089.34
8	C_18:1 n-9_	*Oleic acid*	60,263.38
9	C_18:2 n-6_	*Linoleic acid (LA)*	15,513.28
10	C_18:3 n-3_	*α-linolenic acid (ALA)*	73.64
11	C_18:3 n-6_	*γ-linolenic acid*	1,487.13
12	C_20:0_	*Arachidic acid*	74.07
13	C_20:1 n-9_	*Gondoic acid (Eicosenoic acid)*	649.03
14	C_20:3 n-3_	*Eicosatrienoic acid*	42.65
15	C_20:3 n-6_	*Dihomoγ-linoleic acid (DGLA)*	229.37
16	C_20:4 n-6_	*Arachidonic acid (AA)*	971.30
17	C_20:5 n-3_	*Eicosapentaenoic acid (EPA)*	967.42
18	C_22:5 n-6_	*Osbond acid (Docosapentaenoic acid, DPA n-6)*	894.76
19	C_22:6 n-3_	*Cervonic acid (Docosahexaenoic acid, DHA)*	5,255.59

g – gram; µg – microgram.

### Fatty acid profile of Tukong chicken meat

The fatty acid composition of Tukong chicken meat is detailed in Table 4. The lipid fraction was largely composed of saturated fatty acids (SFAs), particularly dodecanoic acid (C12:0; 46,724.11 µg/g) and tetradecanoic acid (C14:0; 17,690.63 µg/g). Palmitic acid (C16:0; 14,352.74 µg/g) and stearic acid (C18:0; 5,532.84 µg/g) were also present in substantial amounts.

**Table 4:** Fatty acid content of Tukong chicken meat.

No	Code	Fatty Acid	Concentration (µg/g)
1	C_6:0_	*Hexanoic acid*	898.65
2	C_8:0_	*Octanoic acid*	6,919.98
3	C_10:0_	*Decanoic acid*	3,695.03
4	C_12:0_	*Dodecanoic acid*	46,724.11
5	C_14:0_	*Tetradecanoic acid*	17,690.63
6	C_14:1_	*9-Tetradecenoic acid*	218.50
7	C_15:0_	*Pentadecanoic acid*	107.89
8	C_16:0_	*Hexadecanoic acid*	14,352.74
9	C_16:1 n-7_	*9-Hexadecenoic acid*	3,747.88
10	C_17:0_	*Heptadecanoic acid*	107.07
11	C_18:0_	*Octadecanoic acid*	5,532.84
12	C_18:1 n-7_	*11-Octadecenoic acid*	222.19
13	C_18:1 n-9_	*trans-9-Octadecenoic acid*	406.21
14	C_18:1 n-9_	*cis-9-Octadecenoic acid*	26,755.98
15	C_18:2 n-6_	*cis, cis-9,12-Octadecadienoic acid*	5,934.99
16	C_18:2 n-6_	*trans, trans-9,12-Octadecadienoic acid*	370.38
17	C_18:3 n-3_	*cis, cis, cis-9,12,15-Octadecatrienoic acid*	451.79
18	C_18:3 n-6_	*cis, cis, cis-6,9,12-Octadecatrienoic acid*	103.92
19	C_20:0_	*Eicosanoic acid*	339.64
20	C_20:1 n-9_	*11-Eicosenoic acid*	314.84
21	C_20:2 n-6_	*cis-11,14-Eicosadienoic acid*	283.27
22	C_20:3 n-6_	*cis, cis, cis-8,11,14-Eicosatrienoic acid*	197.67
23	C_20:4 n-6_	*cis, cis, cis, cis-5,8,11,14-Eicosatetraenoic acid*	740.97

g – gram; µg – microgram.

Among monounsaturated fatty acids (MUFAs), cis-9-octadecenoic acid (C18:1 n-9; 26,755.98 µg/g) was the predominant component. Linoleic acid (C18:2 n-6; 5,934.99 µg/g) represented the principal polyunsaturated fatty acid fraction, followed by arachidonic acid (C20:4 n-6; 740.97 µg/g). Trace amounts of short- and medium-chain fatty acids (e.g., C6:0 and C8:0) were also detected. In general, Tukong chicken meat demonstrated a lipid profile characterised by elevated saturated fatty acid content, with moderate contributions from monounsaturated and polyunsaturated fatty acids.

## Discussion

The egg index value (>76%) indicates that Tukong chicken eggs possess a relatively round morphology. In terms of structural properties, a rounder egg shape improves mechanical resistance by evenly distributing external pressure across the shell surface, reducing the risk of cracking during handling and transportation. A connection between increased egg index and longer shell durability has been observed in poultry breeding studies (Philippe et al., 2020). Egg index measurements can be utosed in plan breeding and production strategies aimed at enhancing egg physical resilience and improving hatching success rates (Gervais et al., 2016). Geometry has also been shown to affect hatchability, with egg shape potentially influencing internal gas exchange and egg positioning. However, earlier studies have shown contradictory relationships between egg shape and early embryonic mortality; thus, it is not a theory that the geometry of an egg determines incubation success (Alaşahan and Copur, 2016; Isnaini et al., 2023).

Shell thickness and weight are mainly determined by calcium carbonate deposition during shell formation in the uterus. Calcium metabolism, phosphorus balance, and mineral availability are crucial in the formation of shells, as calcium carbonate is found in eggshells at a concentration of approximately 95-96% (Skrivan et al., 2016; Saki et al., 2019). In terms of biology, shell thickness is a measure of the effectiveness and rapidity of mineral mobilization from medullary bone and dietary sources during the egg-laying cycle. Variability in shell quality has been found to be influenced by age, genetics, dietary mineral balance, stress, and health status according to previous studies (Hargitai et al., 2011; Ahmed et al., 2022). Consequently, the shell thickness of Tukong eggs appears relatively consistent, suggesting that mineral deposition has been stable under the present management regime.

The shell color of Tukong chicken eggs ranged from bone white to brownish (Fig. 2). Eggshell coloration is primarily determined by biliverdin and protoporphyrin pigments. Biliverdin, synthesized in the shell gland, is responsible for blue coloration as observed in certain breeds such as Araucana, whereas protoporphyrin contributes to brown pigmentation (Wang et al., 2011). The deposition of these pigments occurs in the shell gland during egg formation, influenced by metabolic activity and blood flow. Genetic factors play a significant role in pigment deposition, including loci such as the oocyan (O) gene associated with blue egg production (Wang et al., 2013). While Tukong eggs did not exhibit blue coloration, the observed variation suggests genetic control over pigment intensity.

The Haugh Unit (HU) is widely used as an indicator of albumen freshness and structural integrity. The observed HU value (71.73 ± 4.66) corresponds to AA-quality eggs according to standard classifications, where >72 is AA, 60–72 is A, 31–60 is B, and <31 is C (Mountney, 2012). Higher HU values indicate superior albumen quality and freshness (Selim et al., 2018). HU values are influenced by several factors, including storage duration, hen age, nutrition, health status, antioxidant supplementation (vitamin C and/or vitamin E), and molting (Robert, 2004; Hesasaga et al., 2020). Elevated HU values are associated with strong ovomucin–lysozyme interactions that maintain albumen height and viscosity. Reductions in HU are typically linked to prolonged storage, protein degradation, and carbon dioxide loss (Hesasaga et al., 2020). The present findings indicate that Tukong eggs possess adequate albumen integrity under the current production conditions.

Yolk characteristics are influenced by both biological and nutritional factors. Egg size is largely determined by yolk and albumen proportions (Rajkumar et al., 2009). Yolk weight is affected by lipid and protein deposition, ovarian development, feed nutrient composition, age, and environmental conditions. Yolk quality is assessed based on color, texture, chewiness, and aroma (Bain et al., 2016).

**Fig. 3:**
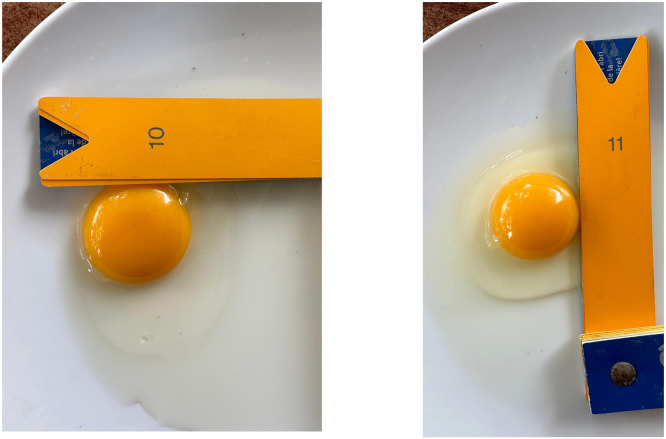
The measurements of egg yolk of the Tukong chicken.

Carotenoid deposition from feed is observed in the developing oocyte, which is why they appear yellow.' After being taken in by the intestine, carotenoids such as lutein and zeaxanthin are then transported to the yolk lipid fraction and stored in the eggshell. The moderate yolk colour score observed in this study is likely a result of the availability of feed pigment rather than differences in genetic pigmentation. Although genetic background and physiological status are factors, previous research indicates that dietary carotenoid concentration is the primary determinant of yolk colour intensity (Kljak et al., 2021). Consumer preferences and yolk pigmentation are also linked, influencing product acceptance. The color of the egg yolk of Tukong chickens is presented in Fig. 3. The color of egg yolks, with a more concentrated hue, attracts the attention not only of household consumers but also of the processed food industry, as they are considered to have higher quality and nutritional content (Çayan and Erener, 2015). The intensity of the yolk color also serves as another indicator of internal quality. The results revealed a correlation between the color value of egg yolk and freshness parameters, such as *the haugh unit (HU),* which measures the quality of albumen or egg white (Sözcü et al., 2021).

The egg and muscle tissues had marked differences in their composition. Tissue-specific nutrient distribution is the biological interpretation of this. The higher protein content in meat is a result of skeleton muscle's primary role as primarily skeletal and contractile protein reservoirs. Conversely, the purpose of eggs is to facilitate embryonic development, resulting in increased moisture content and even lipid-protein interactions (Sözcü et al., 2021). During oogenesis, calcium and phosphorus concentrations indicate the components of embryonic bones and the deposition of physiological nutrients. Avian reproductive physiology is supported by the specific differences observed in the matrix, which have been extensively reported in poultry studies (Sözcü et al., 2021).

To position the present findings within a broader production context, the nutritional composition of Tukong chickens was compared with published data from commercial broilers and indigenous breeds. The protein and fat content of Tukong meat (protein 19.74%, fat 18.15%) was higher than that reported for commercial broilers (protein 17.4%, fat 16%; Mire et al., 2017), Kampung chickens (protein 17.1%, fat 2.43%; Soriano-Santos, 2010), and Sentul chicken (protein 15%, fat 5.3%; Masito et al., 2019). This suggests that Tukong chickens may possess relatively dense muscle protein deposition compared to commonly reported breeds. These findings indicate that Tukong meat may be relatively lean compared to that of commercial broilers. Regarding egg composition, Tukong eggs had lower protein content (11.23%) than that reported for broiler eggs (13%; Nova et al., 2014) and native eggs (16.3%; Helendra et al., 2011). Conversely, the fat content in Tukong eggs (13.98%) was substantially higher than that reported for broiler and native eggs (11.5–12%). While differences in analytical methods and moisture correction may contribute to variation, the observed pattern suggests a potentially favorable protein-to-lipid ratio in Tukong eggs.

**Fig. 4:**
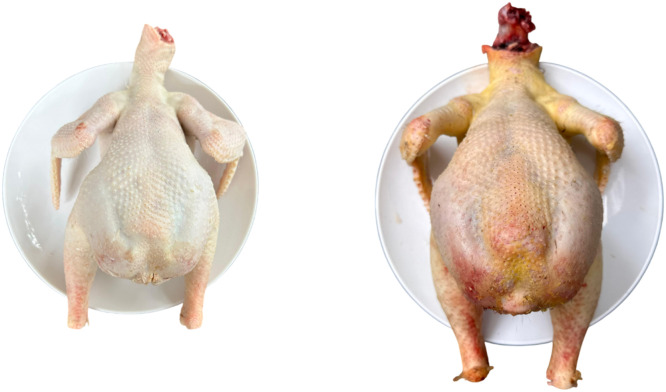
Carcass of the Tukong chicken.

Tukong chickens, including *rumpless* chicken clumps, are known to have a greater percentage of carcasses than normal chickens do, although they tend to weigh less. In the study reported by Tribudi et al. (2023), carcass yield of Tukong chicken was numerically higher than values typically reported for local indigenous chickens (65–72%) and approached the lower range of commercial broilers (70–75%), suggesting a potentially favorable meat-to-body weight ratio under the studied conditions. This is caused by the loss of the tail parts, including the tailbone (*pygostyle*), tail feathers, and *uropygial* glands, which are noncarcass or unconsumed body parts. In the absence of these parts, the total weight of the chicken's body is indeed reduced, but the carcass or meat that can be consumed remains relatively intact. In addition, owing to the absence of energy requirements to form the tail and its supporting structures, the energy and nutrients in the body of *rumpless* chickens are likely to be allocated more to the growth of muscles, such as those in the chest and thighs, resulting in a larger proportion of meat. The results also revealed higher egg production in Tukong chickens, suggesting a more efficient metabolic process and a greater allocation of energy to vital functions. Visually, the observation results (Fig. 4) show that Tukong chickens have a wider, thicker chest, which contributes to improved carcass composition. *Rumpless* chickens tend to have a denser and more compact body shape, which can increase the growth efficiency of body parts that fall into the carcass category (chest, thighs, wings). This results in a carcass that is proportionally larger than that of *non-rumpless* chickens. However, to date, no controlled comparative studies have conclusively demonstrated that rumplessness itself enhances muscle deposition or carcass efficiency, and previous studies on rumpless breeds such as Araucana have primarily focused on egg color rather than carcass composition.

The saturated fatty acid content in eggs suggests that eggs are also a source of energy-dense fats that should be consumed in moderation. Moreover, the unsaturated fat content in Tukong chicken eggs can be categorized into two types: *monounsaturated fatty acids* (MUFAs) and *polyunsaturated fatty acids* (PUFAs). PUFAs have two or more double bonds and play important roles in the development of brain function, cell growth, and the regulation of the immune system. Linoleic acid, palmitoleic acid, and stearic acid are among the key fats found in food sources. Compared with those of Cemani and White Leghorn chickens reported by Kostaman et al. (2021), Tukong eggs exhibit a distinct proximate profile, characterized by elevated protein concentration and reduced lipid levels. While fatty acid class distribution in indigenous and commercial breeds generally falls within similar ranges (SFA 20–35%, MUFA 35–45%, PUFA 10–30%), the markedly lower total lipid fraction in Tukong eggs may influence the absolute fatty acid intake per serving rather than relative distribution. This suggests that Tukong eggs may represent a leaner alternative without substantially altering fatty acid class balance.

Oleic and palmitic acids are the most frequently present in eggs, which aligns with hepatic lipid synthesis patterns in laying hens. In birds, the liver is responsible for lipid production and modification, which are then transported to the developing yolk via very-low-density lipoproteins. Stearoyl-CoA desaturase activity is responsible for the significant amount of stearic acid's conversion into oleic acid. This is due to its high concentration of this substance. DHA and EPA, which are polyunsaturated fatty acids, indicate the movement of necessary essential lipids into the yolk, where they play a crucial role in embryonic neural development. Meat may contain more saturated fatty acids, which can indicate the composition of the muscles' lipids and energy storage patterns. Fatty acid deficiency in muscle is influenced by dietary, genetic, and metabolic factors.

The absence of the pygostyle is indicated by its lack of a central nervous system structure (rumpless trait). Despite the potential for anatomical changes to alter carcass proportions, there is no conclusive evidence to support the direct causal relationship between rumplessness and fatty acid metabolism, as observed in some chicken breeds, such as Tukong and Araucana. The fatty acid content in meat and eggs is strongly influenced by genetic, nutritional, and environmental factors (Wang et al., 2021, 2018). It is probable that any metabolic impact would be mediated by genetic linkages that affect nutrient allocation or growth. Phenotypic differences in carcass yield are evident, but definitive conclusions on fatty acid modulation require controlled comparative studies between rumpless and non-rumpless genotypes. However, there is no evidence to support these findings yet.

## Conclusion

To sum up, Tukong chicken eggs were found to have satisfactory internal quality, which included acceptable Haugh Unit values, uniform shell thickness, and balanced physicochemical characteristics. Protein concentration was higher in muscle and lower in egg, suggesting distinct differences in their composition compared to meat tissues. These findings highlight the nutritional and functional value of Tukong chicken products, a rare indigenous genetic resource. However, this study was limited to a single conservation flock under the same management conditions and excluded comparative genotypes. To better understand the effects of genotypes on metabolic mechanisms, larger populations and controlled comparative designs are required for future studies.

## CRediT authorship contribution statement

**Aprilianna Putri Zahara Nafsina Luvita Sari:** Writing – review & editing, Writing – original draft, Project administration, Methodology, Funding acquisition. **Yuli Arif Tribudi:** Writing – original draft, Supervision, Software, Methodology, Formal analysis, Data curation, Conceptualization. **Yasin Pradana Maulana:** Writing – original draft, Methodology, Formal analysis, Data curation, Conceptualization. **Maria Ulfah:** Writing – review & editing, Writing – original draft, Validation, Supervision. **Danung Nur Adli:** Writing – review & editing, Writing – original draft, Validation, Software, Formal analysis.

## Disclosures

The authors declare that they have no known competing financial interests or personal relationships that could have appeared to influence the work reported in this paper.
